# 1-Chloro-4-(3,4-dichloro­phen­yl)-3,4-dihydro­naphthalene-2-carbaldehyde

**DOI:** 10.1107/S160053681100105X

**Published:** 2011-01-15

**Authors:** H. C. Devarajegowda, P. Nagendra, S. Jeyaseelan, N. Chidananda, Boja Poojary

**Affiliations:** a Department of Physics, Yuvaraja’s College (Constituent College), University of Mysore, Mysore 570 005, Karnataka, India; b Department of Chemistry, Bharathi College, Bharathinagar 571 422, Mandya District, Karnataka, India; c Department of Chemistry, Mangalore University, Mangalagangotri 574 199, Karnataka, India

## Abstract

The title compound, C_17_H_11_Cl_3_O, was synthesized *via* the Vilsmeier–Haack reaction. The dihydro­naphthalene ring system is non-planar, the dihedral angle between the two fused rings being 10.87 (13)°; it forms a dihedral angle of 81.45 (10)° with the dichloro­phenyl ring. The crystal structure features inter­molecular C—H⋯O hydrogen bonds.

## Related literature

For general background to 4-(3,4-dichloro­phen­yl)-3,4- dihydro­naphthalen-1(2*H*)-one, see: Zhengxu *et al.* (2007 ▶); Jerussi *et al.* (2004 ▶); Taber *et al.* (2004 ▶); Ray *et al.* (2003 ▶); Meth-Cohn & Stanforth (1991 ▶); Hurd & Webb (1941 ▶); Mallegol *et al.* (2005 ▶). For the synthesis, see Vilsmeier *et al.* (1937 ▶). For a related structure, see: Gowda *et al.* (2008 ▶).
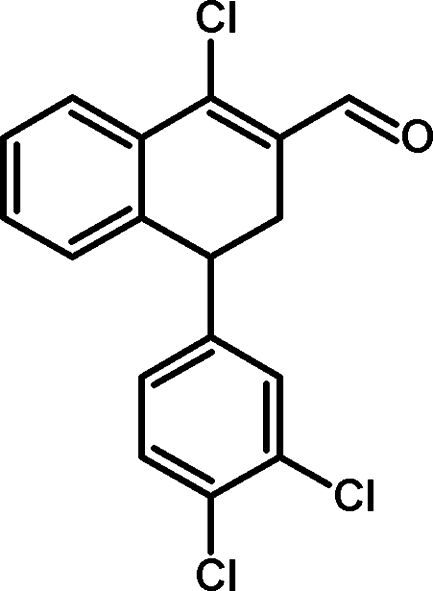


## Experimental

### 

#### Crystal data

C_17_H_11_Cl_3_O
*M* *_r_* = 337.61
Monoclinic, 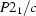

*a* = 10.2969 (5) Å
*b* = 10.8849 (5) Å
*c* = 13.6144 (7) Å
β = 91.436 (5)°
*V* = 1525.43 (13) Å^3^
*Z* = 4
Mo *K*α radiation
μ = 0.60 mm^−1^
*T* = 293 K
0.22 × 0.15 × 0.12 mm

#### Data collection

Oxford Diffraction Xcalibur diffractometer
Absorption correction: multi-scan (*CrysAlis PRO RED*; Oxford Diffraction, 2010 ▶) *T*_min_ = 0.546, *T*_max_ = 1.000
15902 measured reflections
3006 independent reflections
2143 reflections with *I* > 2σ(*I*)
*R*_int_ = 0.043

#### Refinement

*R*[*F* ^2^ > 2σ(*F* ^2^)] = 0.050
*wR*(*F* ^2^) = 0.149
*S* = 1.09
3006 reflections
234 parameters
H atoms treated by a mixture of independent and constrained refinement
Δρ_max_ = 0.52 e Å^−3^
Δρ_min_ = −0.33 e Å^−3^

### 

Data collection: *CrysAlis PRO CCD* (Oxford Diffraction, 2010 ▶); cell refinement: *CrysAlis PRO CCD*; data reduction: *CrysAlis PRO RED* (Oxford Diffraction, 2010 ▶); program(s) used to solve structure: *SHELXS97* (Sheldrick, 2008 ▶); program(s) used to refine structure: *SHELXL97* (Sheldrick, 2008 ▶); molecular graphics: *ORTEP-3* (Farrugia, 1997 ▶) and *CAMERON* (Watkin *et al.*, 1993 ▶); software used to prepare material for publication: *WinGX* (Farrugia, 1999 ▶).

## Supplementary Material

Crystal structure: contains datablocks I, global. DOI: 10.1107/S160053681100105X/bv2168sup1.cif

Structure factors: contains datablocks I. DOI: 10.1107/S160053681100105X/bv2168Isup2.hkl

Additional supplementary materials:  crystallographic information; 3D view; checkCIF report

## Figures and Tables

**Table 1:** Hydrogen-bond geometry (Å, °)

*D*—H⋯*A*	*D*—H	H⋯*A*	*D*⋯*A*	*D*—H⋯*A*
C18—H18⋯O4^i^	0.90 (3)	2.58 (3)	3.201 (3)	128 (2)

Symmetry code: (i) 
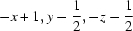
.

**Fig. 1.**
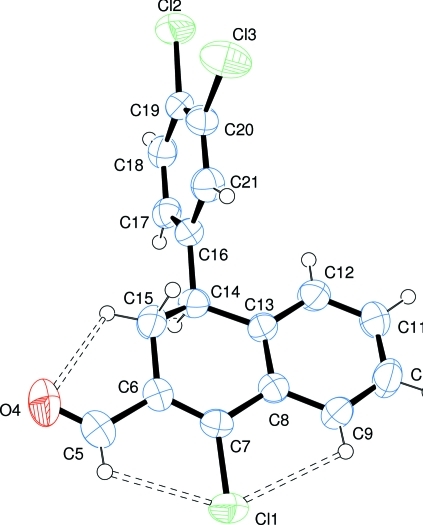
The molecular structure of the title compound. Displacement ellipsoids are drawn at the 50% probability level.Dashed lines indicate hydrogen bonds

**Fig. 2.**
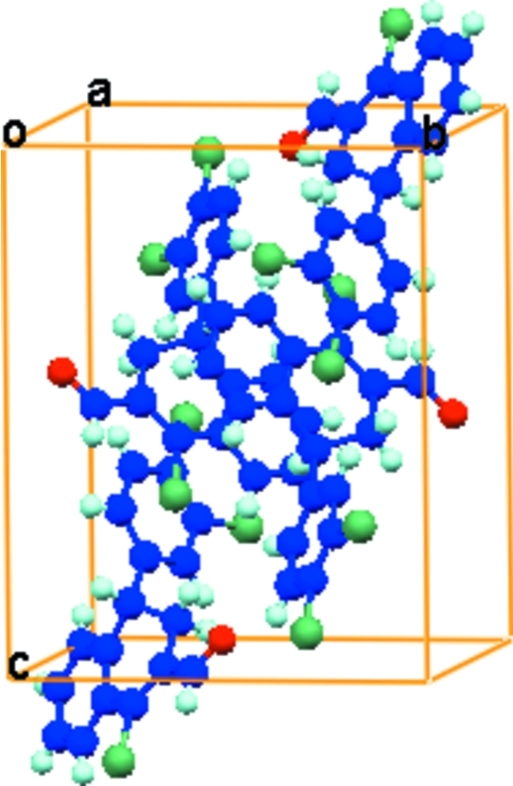
A view of the structure down the axis a.

## supplementary crystallographic information

### Comment

Recently drug candidates for blocking the monoamine reuptake trasporters have considerable interest in the pharmaceutical industry for treatment of central nervous system disorders (Zhengxu *et al.*, 2007). 1,2,3,4-tetrahydronaphthalene derivatives are for the treatment of central nervous system disorders (Jerussi *et al.*, 2004; Taber *et al.*, 2004). Tetrahydronaphthalene derivatives are also used in liquid crystal display elements (Ray *et al.*, 2003). Potent pharmaceutically active 1-chloro-4-(3,4-dichlorophenyl)-3, 4-dihydronaphthalene-2-carbaldehyde was prepared by the Vilsmeier- Haack reaction (Vilsmeier *et al.*, 1937; Meth-Cohn *et al.* , 1991; Hurd *et al.*, 1941; Mallegol *et al.*, 2005) of 4-(3,4-dichlorophenyl)-3,4-dihydronaphthalen-1(2*H*)-one.

The asymmetric unit of the 1-chloro-4-(3,4-dichlorophenyl)-3,4- dihydronaphthalene-2-carbaldehyde contains one molecule (Fig. 1). The dihydronaphthalene ring system is non-planar; the dihedral angle between the two ring system of the naphthalene ring is 10.87 (13)° and also the dihedral angle between the dihydronaphthalene ring system and the dichlorophenyl ring is 81.45 (10)°. The crystal structure shows intramolecular C5—H5···Cl1, C9—H9···Cl1, C15—H15B···O4 and C18—H18···O4 intermolecular hydrogen bonds. Bond distances within the aromatic rings are in agreement with those observed related structures (Gowda *et al.*, 2008). The packing of the molecules shows when viewed along the *a* axis (Fig.2).

### Experimental

To the Vilsmeier-Haack complex prepared from DMF and POCl_3_ (0.03 mol) at 0°C, the compound 4-(3,4-dichlorophenyl)-3,4-dihydronaphthalen-1(2*H*)-one (0.01 mol) was added and the reaction mixture was stirred at 65°C for 4 h. The reaction completion was monitored by TLC. The contents were cooled, poured in to ice-cold water and neutralized using Na_2_CO_3_ solution. The product that separated was filtered and dried. X-ray quality crystals were obtained from an ethyl acetate solution.

### Refinement

Hydrogen atoms were located in a difference Fourier map and were allowed to refine isotropically.

### Crystal data

**Table:**

C_17_H_11_Cl_3_O	*F*(000) = 688
*M**_r_* = 337.61	*D*_x_ = 1.470 Mg m^−^^3^
Monoclinic, *P*2_1_/*c*	Melting point: 383 K
Hall symbol: -P 2ybc	Mo *K*α radiation, λ = 0.71073 Å
*a* = 10.2969 (5) Å	Cell parameters from 3006 reflections
*b* = 10.8849 (5) Å	θ = 2.4–26.0°
*c* = 13.6144 (7) Å	µ = 0.60 mm^−^^1^
β = 91.436 (5)°	*T* = 293 K
*V* = 1525.43 (13) Å^3^	Plate, colourless
*Z* = 4	0.22 × 0.15 × 0.12 mm

### Data collection

**Table:**

Oxford Diffraction Xcalibur diffractometer	3006 independent reflections
Radiation source: Enhance (Mo) X-ray Source	2143 reflections with *I* > 2σ(*I*)
graphite	*R*_int_ = 0.043
Detector resolution: 16.0839 pixels mm^-1^	θ_max_ = 26.0°, θ_min_ = 2.4°
ω scans	*h* = −12→12
Absorption correction: multi-scan (*CrysAlis PRO RED*; Oxford Diffraction, 2010)	*k* = −13→13
*T*_min_ = 0.546, *T*_max_ = 1.000	*l* = −16→16
15902 measured reflections	

### Refinement

**Table:**

Refinement on *F*^2^	Primary atom site location: structure-invariant direct methods
Least-squares matrix: full	Secondary atom site location: difference Fourier map
*R*[*F*^2^ > 2σ(*F*^2^)] = 0.050	Hydrogen site location: inferred from neighbouring sites
*wR*(*F*^2^) = 0.149	H atoms treated by a mixture of independent and constrained refinement
*S* = 1.09	*w* = 1/[σ^2^(*F*_o_^2^) + (0.0885*P*)^2^] where *P* = (*F*_o_^2^ + 2*F*_c_^2^)/3
3006 reflections	(Δ/σ)_max_ = 0.001
234 parameters	Δρ_max_ = 0.52 e Å^−^^3^
0 restraints	Δρ_min_ = −0.33 e Å^−^^3^

### Special details

**Table:**

Experimental. *CrysAlis PRO*, Oxford Diffraction Ltd., Version 1.171.33.55 (release 05–01–2010 CrysAlis171. NET) Empirical absorption correction using spherical harmonics, implemented in SCALE3 ABSPACK scaling algorithm.^1^H NMR (CDCl_3_, 400 MHz): δ, 10.33 (s, 1H, -CHO), 6.92-8.00 (m, 7H, Ar-H), 4.13 (t, 1H, –CH proton of fused cyclohexane ring, J=10.0 Hz), 2.86-3.01(m, 2H, –CH_2_ proton of fused cyclohexane ring)IR (KBr, cm^-1^): 3443.28 (-CHO), 1662.34 (C=O of aldehyde), 1595.81(C=C,aromatic), 838.883 (C-Cl), 1255.43 (C-H stretch).FAB MASS: m/z = 337, mol. formulae: C_17_H_11_Cl_3_O).
Geometry. All s.u.'s (except the s.u. in the dihedral angle between two l.s. planes) are estimated using the full covariance matrix. The cell s.u.'s are taken into account individually in the estimation of s.u.'s in distances, angles and torsion angles; correlations between s.u.'s in cell parameters are only used when they are defined by crystal symmetry. An approximate (isotropic) treatment of cell s.u.'s is used for estimating s.u.'s involving l.s. planes.
Refinement. Refinement of *F*^2^ against ALL reflections. The weighted *R*-factor *wR* and goodness of fit *S* are based on *F*^2^, conventional *R*-factors *R* are based on *F*, with *F* set to zero for negative *F*^2^. The threshold expression of *F*^2^ > 2σ(*F*^2^) is used only for calculating *R*-factors(gt) *etc*. and is not relevant to the choice of reflections for refinement. *R*-factors based on *F*^2^ are statistically about twice as large as those based on *F*, and *R*- factors based on ALL data will be even larger.

### Fractional atomic coordinates and isotropic or equivalent isotropic displacement parameters (Å^2^)

**Table:**

	*x*	*y*	*z*	*U*_iso_*/*U*_eq_	
Cl1	0.40177 (8)	0.17987 (7)	0.18417 (6)	0.0642 (3)	
Cl2	1.03593 (8)	0.22018 (9)	−0.40674 (6)	0.0809 (3)	
Cl3	1.07383 (9)	0.35613 (9)	−0.20272 (7)	0.0853 (3)	
O4	0.3558 (2)	0.4375 (2)	−0.04817 (19)	0.0873 (8)	
C5	0.3745 (3)	0.3618 (3)	0.0142 (3)	0.0654 (8)	
C6	0.4835 (3)	0.2751 (2)	0.01503 (19)	0.0461 (6)	
C7	0.5053 (2)	0.1922 (2)	0.08561 (18)	0.0410 (6)	
C8	0.6168 (2)	0.1084 (2)	0.08586 (17)	0.0389 (6)	
C9	0.6561 (3)	0.0405 (2)	0.16846 (19)	0.0474 (6)	
C10	0.7619 (3)	−0.0361 (3)	0.1655 (2)	0.0604 (8)	
C11	0.8291 (3)	−0.0479 (3)	0.0801 (2)	0.0595 (8)	
C12	0.7912 (3)	0.0171 (2)	−0.0023 (2)	0.0520 (7)	
C13	0.6860 (2)	0.0965 (2)	−0.00149 (17)	0.0409 (6)	
C14	0.6365 (3)	0.1633 (2)	−0.09391 (19)	0.0462 (6)	
C15	0.5787 (3)	0.2861 (3)	−0.0664 (2)	0.0539 (7)	
C16	0.7379 (2)	0.1794 (2)	−0.17227 (19)	0.0433 (6)	
C17	0.7223 (3)	0.1248 (2)	−0.26254 (19)	0.0447 (6)	
C18	0.8128 (3)	0.1384 (2)	−0.3336 (2)	0.0475 (6)	
C19	0.9213 (3)	0.2083 (2)	−0.31643 (19)	0.0451 (6)	
C20	0.9393 (3)	0.2661 (2)	−0.2272 (2)	0.0476 (6)	
C21	0.8493 (3)	0.2513 (2)	−0.1548 (2)	0.0503 (7)	
H5	0.327 (3)	0.359 (3)	0.067 (2)	0.080 (11)*	
H9	0.616 (3)	0.050 (2)	0.224 (2)	0.061 (8)*	
H10	0.792 (3)	−0.082 (3)	0.218 (2)	0.088 (11)*	
H11	0.904 (3)	−0.096 (2)	0.0799 (18)	0.050 (7)*	
H12	0.836 (3)	0.009 (2)	−0.054 (2)	0.057 (8)*	
H14	0.561 (2)	0.111 (2)	−0.1275 (17)	0.040 (6)*	
H17	0.653 (3)	0.068 (2)	−0.2739 (18)	0.049 (7)*	
H18	0.807 (3)	0.103 (2)	−0.393 (2)	0.055 (8)*	
H15A	0.668 (4)	0.339 (3)	−0.040 (3)	0.101 (12)*	
H15B	0.536 (3)	0.324 (2)	−0.121 (2)	0.057 (8)*	
H21	0.867 (3)	0.291 (3)	−0.090 (2)	0.061 (8)*	

### Atomic displacement parameters (Å^2^)

**Table:**

	*U*^11^	*U*^22^	*U*^33^	*U*^12^	*U*^13^	*U*^23^
Cl1	0.0575 (5)	0.0748 (5)	0.0613 (5)	0.0080 (3)	0.0232 (4)	0.0094 (4)
Cl2	0.0618 (5)	0.1218 (8)	0.0600 (5)	0.0032 (5)	0.0216 (4)	0.0190 (5)
Cl3	0.0590 (5)	0.0868 (6)	0.1103 (8)	−0.0339 (4)	0.0057 (5)	−0.0100 (5)
O4	0.0824 (17)	0.0849 (16)	0.0945 (18)	0.0370 (13)	−0.0004 (14)	0.0272 (14)
C5	0.0517 (19)	0.073 (2)	0.072 (2)	0.0171 (15)	0.0058 (17)	0.0092 (18)
C6	0.0425 (14)	0.0474 (14)	0.0484 (15)	0.0052 (11)	−0.0004 (12)	0.0029 (11)
C7	0.0372 (13)	0.0438 (13)	0.0421 (13)	−0.0044 (11)	0.0042 (11)	−0.0034 (11)
C8	0.0386 (13)	0.0339 (12)	0.0442 (13)	−0.0052 (10)	0.0001 (11)	−0.0009 (10)
C9	0.0547 (17)	0.0480 (14)	0.0396 (14)	0.0000 (12)	0.0037 (13)	0.0049 (12)
C10	0.071 (2)	0.0574 (17)	0.0522 (17)	0.0116 (15)	−0.0045 (16)	0.0149 (14)
C11	0.0551 (18)	0.0571 (17)	0.0664 (19)	0.0192 (14)	0.0034 (15)	0.0116 (14)
C12	0.0526 (17)	0.0517 (15)	0.0521 (17)	0.0102 (13)	0.0116 (14)	0.0049 (13)
C13	0.0409 (13)	0.0389 (13)	0.0430 (13)	−0.0027 (10)	0.0021 (11)	0.0042 (10)
C14	0.0415 (14)	0.0513 (15)	0.0458 (14)	−0.0039 (12)	0.0028 (12)	0.0036 (12)
C15	0.0569 (18)	0.0555 (16)	0.0496 (16)	0.0149 (14)	0.0075 (14)	0.0144 (13)
C16	0.0392 (14)	0.0436 (13)	0.0472 (15)	0.0002 (11)	0.0037 (11)	0.0098 (11)
C17	0.0467 (15)	0.0380 (13)	0.0493 (15)	−0.0048 (11)	−0.0035 (12)	0.0064 (11)
C18	0.0510 (16)	0.0484 (14)	0.0430 (15)	0.0036 (12)	−0.0020 (12)	0.0014 (12)
C19	0.0425 (14)	0.0483 (14)	0.0446 (14)	0.0042 (11)	0.0053 (12)	0.0112 (12)
C20	0.0395 (14)	0.0460 (14)	0.0570 (17)	−0.0077 (11)	−0.0005 (12)	0.0046 (12)
C21	0.0529 (16)	0.0534 (15)	0.0444 (15)	−0.0016 (12)	−0.0017 (13)	−0.0031 (13)

### Geometric parameters (Å, °)

**Table:**

Cl1—C7	1.740 (2)	C12—C13	1.386 (3)
Cl2—C19	1.731 (2)	C12—H12	0.86 (3)
Cl3—C20	1.722 (3)	C13—C14	1.530 (3)
O4—C5	1.195 (4)	C14—C15	1.514 (4)
C5—C6	1.466 (4)	C14—C16	1.521 (3)
C5—H5	0.88 (3)	C14—H14	1.06 (2)
C6—C7	1.333 (3)	C15—H15A	1.14 (4)
C6—C15	1.503 (3)	C15—H15B	0.94 (3)
C7—C8	1.465 (3)	C16—C17	1.371 (4)
C8—C9	1.398 (3)	C16—C21	1.404 (4)
C8—C13	1.408 (3)	C17—C18	1.368 (4)
C9—C10	1.373 (4)	C17—H17	0.95 (3)
C9—H9	0.88 (3)	C18—C19	1.367 (4)
C10—C11	1.374 (4)	C18—H18	0.89 (3)
C10—H10	0.92 (3)	C19—C20	1.377 (4)
C11—C12	1.375 (4)	C20—C21	1.379 (4)
C11—H11	0.93 (3)	C21—H21	0.99 (3)
			
O4—C5—C6	123.9 (3)	C16—C14—C13	114.3 (2)
O4—C5—H5	121 (2)	C15—C14—H14	106.9 (12)
C6—C5—H5	115 (2)	C16—C14—H14	105.6 (12)
C7—C6—C5	123.8 (2)	C13—C14—H14	109.1 (12)
C7—C6—C15	119.0 (2)	C6—C15—C14	112.3 (2)
C5—C6—C15	117.1 (2)	C6—C15—H15A	110.4 (19)
C6—C7—C8	122.7 (2)	C14—C15—H15A	101.8 (18)
C6—C7—Cl1	120.84 (19)	C6—C15—H15B	108.2 (16)
C8—C7—Cl1	116.44 (17)	C14—C15—H15B	111.5 (16)
C9—C8—C13	119.2 (2)	H15A—C15—H15B	113 (2)
C9—C8—C7	122.8 (2)	C17—C16—C21	118.1 (2)
C13—C8—C7	117.9 (2)	C17—C16—C14	120.8 (2)
C10—C9—C8	120.8 (2)	C21—C16—C14	121.2 (2)
C10—C9—H9	119.4 (19)	C18—C17—C16	121.4 (3)
C8—C9—H9	119.7 (19)	C18—C17—H17	118.4 (15)
C9—C10—C11	119.9 (3)	C16—C17—H17	119.8 (15)
C9—C10—H10	124 (2)	C19—C18—C17	120.5 (3)
C11—C10—H10	116 (2)	C19—C18—H18	115.4 (18)
C10—C11—C12	120.2 (3)	C17—C18—H18	124.1 (18)
C10—C11—H11	119.2 (16)	C18—C19—C20	119.8 (2)
C12—C11—H11	120.4 (16)	C18—C19—Cl2	119.3 (2)
C11—C12—C13	121.3 (3)	C20—C19—Cl2	120.9 (2)
C11—C12—H12	117.9 (19)	C19—C20—C21	119.9 (2)
C13—C12—H12	120.7 (19)	C19—C20—Cl3	121.4 (2)
C12—C13—C8	118.5 (2)	C21—C20—Cl3	118.7 (2)
C12—C13—C14	122.3 (2)	C20—C21—C16	120.3 (3)
C8—C13—C14	119.1 (2)	C20—C21—H21	118.3 (17)
C15—C14—C16	110.7 (2)	C16—C21—H21	121.3 (17)
C15—C14—C13	110.0 (2)		
			
O4—C5—C6—C7	178.8 (3)	C12—C13—C14—C16	−24.0 (3)
O4—C5—C6—C15	2.0 (5)	C8—C13—C14—C16	160.9 (2)
C5—C6—C7—C8	−178.5 (3)	C7—C6—C15—C14	34.9 (4)
C15—C6—C7—C8	−1.7 (4)	C5—C6—C15—C14	−148.1 (3)
C5—C6—C7—Cl1	0.6 (4)	C16—C14—C15—C6	−176.7 (2)
C15—C6—C7—Cl1	177.4 (2)	C13—C14—C15—C6	−49.5 (3)
C6—C7—C8—C9	165.8 (3)	C15—C14—C16—C17	−118.7 (3)
Cl1—C7—C8—C9	−13.4 (3)	C13—C14—C16—C17	116.5 (3)
C6—C7—C8—C13	−14.4 (4)	C15—C14—C16—C21	60.7 (3)
Cl1—C7—C8—C13	166.37 (17)	C13—C14—C16—C21	−64.1 (3)
C13—C8—C9—C10	0.8 (4)	C21—C16—C17—C18	0.7 (4)
C7—C8—C9—C10	−179.4 (2)	C14—C16—C17—C18	−179.9 (2)
C8—C9—C10—C11	−1.0 (5)	C16—C17—C18—C19	−0.6 (4)
C9—C10—C11—C12	0.2 (5)	C17—C18—C19—C20	−0.4 (4)
C10—C11—C12—C13	0.6 (5)	C17—C18—C19—Cl2	178.27 (19)
C11—C12—C13—C8	−0.8 (4)	C18—C19—C20—C21	1.3 (4)
C11—C12—C13—C14	−175.9 (3)	Cl2—C19—C20—C21	−177.3 (2)
C9—C8—C13—C12	0.1 (4)	C18—C19—C20—Cl3	−179.4 (2)
C7—C8—C13—C12	−179.7 (2)	Cl2—C19—C20—Cl3	1.9 (3)
C9—C8—C13—C14	175.4 (2)	C19—C20—C21—C16	−1.3 (4)
C7—C8—C13—C14	−4.4 (3)	Cl3—C20—C21—C16	179.5 (2)
C12—C13—C14—C15	−149.2 (3)	C17—C16—C21—C20	0.3 (4)
C8—C13—C14—C15	35.7 (3)	C14—C16—C21—C20	−179.2 (2)

### Hydrogen-bond geometry (Å, °)

**Table:**

*D*—H···*A*	*D*—H	H···*A*	*D*···*A*	*D*—H···*A*
C5—H5···Cl1	0.88 (3)	2.62 (3)	3.053 (4)	111 (2)
C9—H9···Cl1	0.88 (3)	2.66 (3)	3.039 (3)	107 (2)
C15—H15B···O4	0.94 (3)	2.46 (3)	2.841 (4)	103.8 (19)
C18—H18···O4^i^	0.90 (3)	2.58 (3)	3.201 (3)	128 (2)

Symmetry codes: (i) −*x*+1, *y*−1/2, −*z*−1/2.
